# A RISING TIDE LIFTS ALL BOATS: OLDER WORKER EMPLOYMENT RATES

**DOI:** 10.1093/geroni/igz038.2398

**Published:** 2019-11-08

**Authors:** Robert Harootyan

**Affiliations:** Senior Service America Inc., Silver Spring, Maryland, United States

## Abstract

U.S. population aging has been accompanied by aging of its workforce. One-fourth of workers are now 55+, a result of increasing labor force participation rates (LFPRs) of boomers. Older female LFPRs increased notably since 2000, now 34% (1.5 times greater than the 1947-2000 average). Older male LFPRs steadily declined for 50 years, but increased since 2000 (currently 45%). Despite higher LFPRs, many older workers are underemployed, unemployed or having trouble finding desired jobs. We conducted a pilot to test the hypothesis that areas with strong economic and overall-employment growth are more likely to have higher age 55+ LFPRs - i.e., strong overall demand should increase the likelihood that older persons are employed. Major metropolitan areas were selected because of their large labor pools and ACS age-specific labor force data. Of 318 large metros, we selected 14 whose 2017-2018 employment growth ranged from 0.7% to 3.3% (excluded were those with small negative change and those with concentrated special industries). Results: After calculating age-specific LFPRs from ACS five-year data and overall employment growth for each metro, we found a generally strong relationship between high growth and higher than average LFPRs for persons ages 55+. For example, two opposites: Dallas, with 3.2% employment growth, had age 55+ (five year intervals to 75+) LFPRs substantially higher than the national average (by 4-7 percentage points; significant at 0.05). But Pittsburgh, with 0.8% overall employment growth, had age 55+ LFPRs slightly lower than national average. Conclusion: A rising (economic/employment) tide lifts all boats, including older workers.

